# Spectrum of Typical and Atypical Pulmonary CT Imaging Findings of COVID-19 Infection: A Retrospective Study

**DOI:** 10.7759/cureus.23550

**Published:** 2022-03-27

**Authors:** Monika Sharma, Anchal Sharma, Sidhant Lochav, Varsha Gangta, Y S Gulati, Harharpreet Kaur, Aditya Kaul

**Affiliations:** 1 Department of Radiodiagnosis, Maharishi Markandeshwar Medical College and Hospital, Solan, IND; 2 Department of Medicine, Maharishi Markandeshwar Medical College and Hospital, Solan, IND

**Keywords:** hrct of chest, rtpcr, ground-glass opacity, covid-19, pneumonia, coronavirus

## Abstract

Coronavirus disease 2019 (COVID-19) is caused by severe acute respiratory syndrome coronavirus 2 (SARS-CoV-2). The first known case was identified in Wuhan, China, in December 2019. It was declared a public health emergency by WHO in January 2020. The definitive diagnostic test for COVID-19 is a real time polymerase chain reaction test (RT-PCR) which is highly specific, but sensitivity is variable. COVID-19 typically presents clinically with respiratory and systemic symptoms. The majority of the infected patients are asymptomatic during the course of the disease, which we have not included in our study. Imaging findings on high-resolution computed tomography (HRCT) chest are important to diagnose the disease in early stage, for treatment planning and to predict the patient prognosis. The purpose of our study was to characterize typical and atypical pulmonary and extra-pulmonary HRCT findings in patients with COVID-19 infection and to help in the management of patients.

In this retrospective study, we have included 70 patients who had undergone HRCT examination of the chest in the Radiodiagnosis Department, Maharishi Markandeshwar Medical College, Kumarhatti, Solan, Himachal Pradesh, India. The HRCT findings of the chest of these patients in the study will be evaluated and data will be statistically analyzed.

## Introduction

A series of cases of pneumonia [1] of unknown causation emerged in Wuhan, China, in December 2019. A novel bat-origin coronavirus, coronavirus 2019 (COVID-19) was identified by means of deep sequencing analysis. On December 30, 2019, the WHO announced the event constituted as a public health emergency of international concern (PHEIC). The disease was declared a pandemic by the WHO on March 22, 2020. The mode of transmission [2] of the disease is via human-to-human contact, droplets, airborne transmission is also possible in certain conditions.

The COVID-19 pandemic has been connected to severe acute respiratory syndrome coronavirus (SARS-CoV) and Middle East respiratory syndrome coronavirus (MERS-CoV). Severe acute respiratory syndrome coronavirus 2 (SARS-CoV-2) is an enveloped single-stranded RNA virus. Real-time reverse transcriptase polymerase chain reaction (RT-PCR) assay of nasal and pharyngeal swab specimens is currently the gold standard for the diagnosis of COVID-19. The clinical presentation is variable and includes asymptomatic cases, mildly symptomatic cases, fever, cough, anosmia, dyspnea and respiratory distress in its severe form. Based on literature, high-risk patients for developing severe disease are patients with chronic history of hypertension, diabetics and immune-compromised individuals [3]. As of August 2021, the world has witnessed more than 4.3 million deaths among more than 205 million total cases due to the COVID-19 pandemic.

The chest imaging findings of COVID-19 were first published in January 2020 and included bilateral lung involvement and ground glass opacities (GGO) in the majority of hospitalized patients [4]. Imaging plays an important role in the diagnosis of COVID-19 pneumonia. CT is considered the first-line imaging modality in highly suspected cases and is helpful for monitoring imaging changes during treatment and for the follow-up. Therefore, CT has been identified as an efficient clinical diagnostic tool for people with suspected COVID-19 [5].

Chest CT scanning may have a potential role as a problem-solving diagnostic tool in patients in whom RT-PCR testing remains negative, despite persistent clinical suspicion. CT scans that are performed as part of standard clinical care, for reasons other than COVID-19 evaluation (eg, oncologic follow-up CT scans), may reveal lung abnormalities that can suggest the diagnosis of COVID-19, even in asymptomatic individuals [6-9]. The Fleischner Society recently published an expert opinion statement on the use of chest imaging (including radiography and CT scanning) in patient treatment during the COVID-19 pandemic, with the intent to offer guidance to physicians on the use of thoracic imaging across a breadth of health care environments. However, the Fleischner Society also acknowledged that the evidence base that supported the use of imaging across the scenarios presented was scant and that their advice may undergo refinement through rigorous scientific investigation [10,11].

The aim of the present study was to review various typical and atypical chest CT imaging manifestations of SARS-CoV-2 infection in a cohort of RT-PCR positive patients who underwent CT in our institute, and to confirm and add to emerging literature about this novel virus that took the world by storm and to guide the clinicians in triaging and managing patients on the basis of CT findings.

## Materials and methods

This was a retrospective study of 70 patients from December 2020 to May 2021 who underwent HRCT Chest in our institute (which was regional isolation and tertiary Covid care centre). Maharishi Markandeshwar Medical College and Hospital, Solan, India, issued approval MMMCH/IEC/22/507. Various demographic characteristics were noted and the results were analyzed statistically. All statistical analyses were performed by using SPSS statistical software (version 20.0; IBM Corp., Armonk, NY, USA). P values less than 0.05 were considered as statistically significant.

HRCT imaging protocols used was as follows: 1.25mm slice thickness, 0.625mm of detector collimation, 120 kV tube voltage, and 250-300 mA tube current.

Inclusion criteria were RT-PCR positive patients for SARS-CoV-2 admitted to our institute from period of December 2020 to May 2021; Available HRCT imaging data with CT done anytime during the admission (most of our patients were admitted during five to 30 days of COVID-19 infection) and contrast enhanced CT chest imaging data was available for clinically suspected pulmonary thromboembolism and high D-dimer. Exclusion criteria were age less than 18 years.

Imaging interpretation and imaging tools

All CT examinations were assessed by two radiologists independently. The CT patterns were described according to the terms [12] defined by the Fleischner Society and peer-reviewed literature on viral pneumonia. Images were assessed into three broad categories: typical findings, atypical pulmonary and atypical extrapulmonary findings (as defined by the Radiological Society of North America (RSNA) in expert consensus document on reporting CT chest findings) [13].

Typical HRCT findings included ground-glass opacities (hazy areas of increased attenuation without obscuration of the underlying vasculature); organizing consolidation (homogeneous opacification with obscuration of underlying vasculature); mixed pattern (combination of ground glass opacities and organizing consolidation); crazy paving (inter/intra-lobular septal thickening in the regions of increased lung attenuation); reverse halo sign/atoll sign (focal rounded ground glass opacity surrounded by a more or less complete ring-like consolidation); and early to late signs of fibrosis [14] which included reticular pattern (fine network or mesh of overlapping linear lines), architectural distortion (any distortion of the normal lung parenchymal anatomy), tractional bronchiectasis and bronchiolectasis, honeycomb cysts (subpleural clustered cystic air spaces with size 3-5 mm). In our study, for image analysis purpose, early fibrosis was defined as <14 days of onset of symptoms and late fibrosis as >14 days of symptoms onset on HRCT chest.

The distribution of abnormalities were also classified as predominantly subpleural (involving mainly the peripheral one-third of the lung), central (involving mainly the central two-third of the lung), or diffuse.

Atypical pulmonary imaging findings included nodules, cavitatory lesions and cystic air-spaces/air bubble sign in the affected lung.

Atypical extra-pulmonary findings included mediastinal lymph nodes (short axis more than 10 mm), dilated main pulmonary artery (maximum transverse diameter of 27 mm for women and 29 mm for men according to Framingham heart study [15]), pleural effusion, pneumomediastinum, pneumothorax, pulmonary thromboembolism and pericardial effusion.

Images were assessed and also categorized according to CT severity scoring system, presented by Li et al. [16] and published in Investigative Radiology in March 2020. Briefly in this system, both lungs are divided into five lobes, and each lobe assessed individually. Each lobe could be awarded a CT score from 0 to 5, depending on the percentage of the involved lobe: score 0 - 0% involvement; score 1 - less than 5% involvement; score 2 - 5% to 25% involvement; score 3 - 26% to 49% involvement; score 4 - 50% to 75% involvement; score 5 - greater than 75% involvement. The overall CT score is the sum of the points from each lobe and ranges from 0 to 25 points.

For standardization and analysis, all images were categorized into mild (1 to 7), moderate (8 to 14) and severe (15 to 25); according to this CT severity scoring system.

## Results

Among the studied 70 patients, 38 were males (54.28%) and 32 were females (45.71%) with mean age 65.5 years (range 22 to 87 years). All 70 patients were RT-PCR positive and admitted to our hospital for isolation (as our institute was designated as regional isolation and tertiary care centre) and further management.

Forty-four patients were admitted to the ICU and 21 of them had progressive acute respiratory distress syndrome (ARDS)-like symptoms and need for mechanical ventilation during our study period. Out of the 21 patients who needed mechanical ventilation, seven of them died, so the mortality rate in our cohort was 10%. Most of the patients who underwent HRCT were symptomatic and HRCT was performed within five to 30 days of hospital admission (average 10-14 days) and were further categorized according to CT severity scores. In our institute, HRCT was done for all symptomatic COVID-19-positive patients.

As mentioned previously, the imaging findings were categorized as typical pulmonary, atypical pulmonary and atypical extra-pulmonary findings and data was analyzed accordingly (Table 1).

**Table 1:** Incidence of typical and atypical imaging findings. GGO: ground glass opacities, MPA: main pulmonary artery

	CT Imaging Findings	No. of Cases (n=70)
A)	TYPICAL PULMONARY FINDINGS	
1	Only GGO	36(51.42%)
2	Only Organising Consolidation	11(15.71%)
4	Total GGO	58(82.8%)
5	Mixed Pattern (GGO + Organising Consolidation)	22(31.42%)
6	Crazy Paving Pattern	37(52.85%)
7	Reverse Halo Sign	4(5.71%)
8	Fibrosis	61(87.14%)
B)	ATYPICAL PULMONARY FINDINGS	
1	Cavity	4(5.71%)
2	Cystic Air Spaces(Air Bubble Sign)	2(2.85%)
3	Nodules	1(1.42%)
C)	ATYPICAL EXTRAPULMONARY FINDINGS	
1	Pleural Effusion	9(12.85%)
2	Mediastinal Nodes	24(34.28%)
3	Pericardial Effusion	2(2.85%)
4	Dilated MPA	11(15.71%)
5	Pneumomediastinum	3(4.28%)
6	Pulmonary Thromboembolism	1(1.42%)
7	Pneumothorax	1(1.42%)

Typical pulmonary findings, included only GGO in 36 (51.42%), total GGO (in combination with other findings) in 58 (82.85%), organising consolidation in 11 (15.71%), mixed pattern in 22 (31.42%), crazy paving in 37 (52.85%), reverse halo sign in four (5.71%) and fibrosis in 61 (87.14%) patients. Based on this statistical analysis, in our cohort the maximum patients had fibrosis as a predominant HRCT finding followed by GGO.

In our study, atypical pulmonary findings were cavity in four cases (5.71%), followed by cystic air spaces in two (2.85%) cases and nodules in one (1.42%) case.

Atypical extra-pulmonary finding, included mediastinal lymph nodes in 24 (34.28%), dilated main pulmonary artery in 11 (15.71%), pleural effusion in nine (12.85%), pneumo-mediastinum in three (4.28%), pericardial effusion in two (2.85%), pneumothorax in one (1.42%) and pulmonary thrombo-embolism in one (1.42%) patient.

There was significant association of pulmonary fibrosis and dilated pulmonary artery noted with total of 12 patients with HRCT findings of dilated pulmonary artery; 11 also had imaging features of pulmonary fibrosis and the remaining one had background changes of emphysema.

In our study, CT severity score was significantly higher in patients with atypical findings (p value less than 0.05).

## Discussion

In our study, the most common CT finding was fibrosis (reticulations/architectural distortion/tractional bronchiectasis/honeycomb cysts) seen in 61 (87.14%) cases. Pulmonary fibrosis is a known sequela of ARDS and available data indicates about 41.8% of patients with COVID-19 develop ARDS. Pulmonary fibrosis was autopsy proven in the predecessors of SARS-CoV-2, i.e. in SARS and MERS [17,18].

In a study by Muller et al., five out of 15 SARS patients who had ground glass opacity developed a combination of irregular interlobular septal thickening and mild tractional bronchiectasis after two to 17 days of hospital admission [19]. In a study of 62 patients by Zhou et al. [20], fibrotic changes were seen in 21 (33.9%) patients, with this finding more likely to occur in advanced-phase disease (eight to 14 days after the onset of symptoms) than the early phase of the disease (≤seven days after the onset of symptoms).

**Figure 1:**
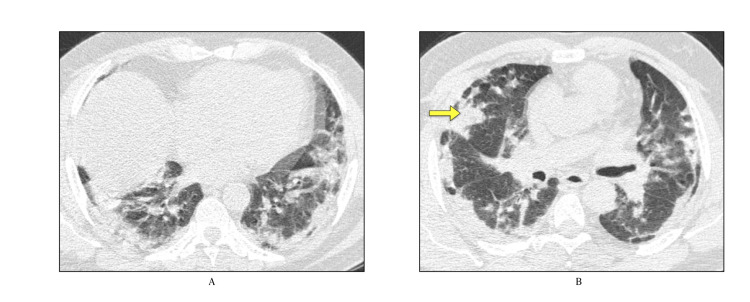
A) and B) Lung window axial sections Areas of organizing consolidation (arrow) with fibrotic changes involving bilateral lungs in subpleural distribution.

As in our study most of the patients had undergone HRCT in later days of disease, fibrosis as the most common finding is not alarming and further supports that in later stages there is significant fibrosis complicating the disease progression. The finding of fibrotic changes this early in the disease suggests an attempt at repair following pulmonary injury [21]. Since many patients were lost to follow-up after discharge, it is not known whether these changes will resolve with time or progress to permanent changes. Fibrotic areas in the subpleural distribution with areas of consolidation are seen (Figure 1).

**Figure 2:**
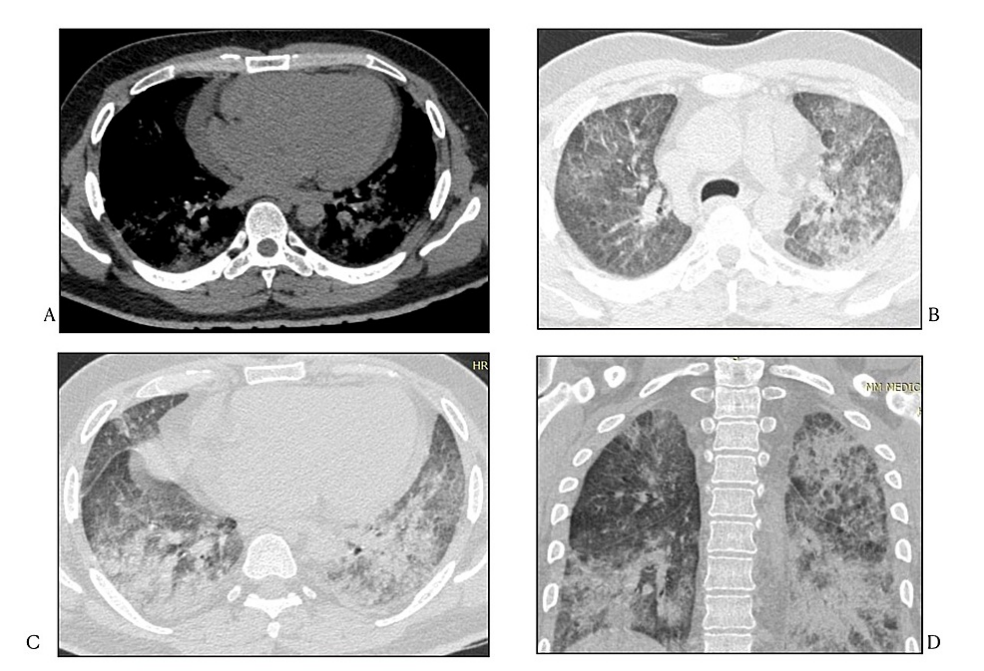
Patient with CT severity score of 21/25 HRCT CHEST (mediastinal and lung windows) axial and coronal reformatted images show diffuse changes of acute respiratory distress syndrome with cardiomegaly and pericardial effusion

An elderly female with COVID-19 infection who presented with severe shortness of breath and chest pain with HRCT severity score of 21/25 (severe) demonstrates diffuse ground glass opacities with both central and subpleural distribution along with interlobular septal thickening (Figure 2).

**Figure 3:**
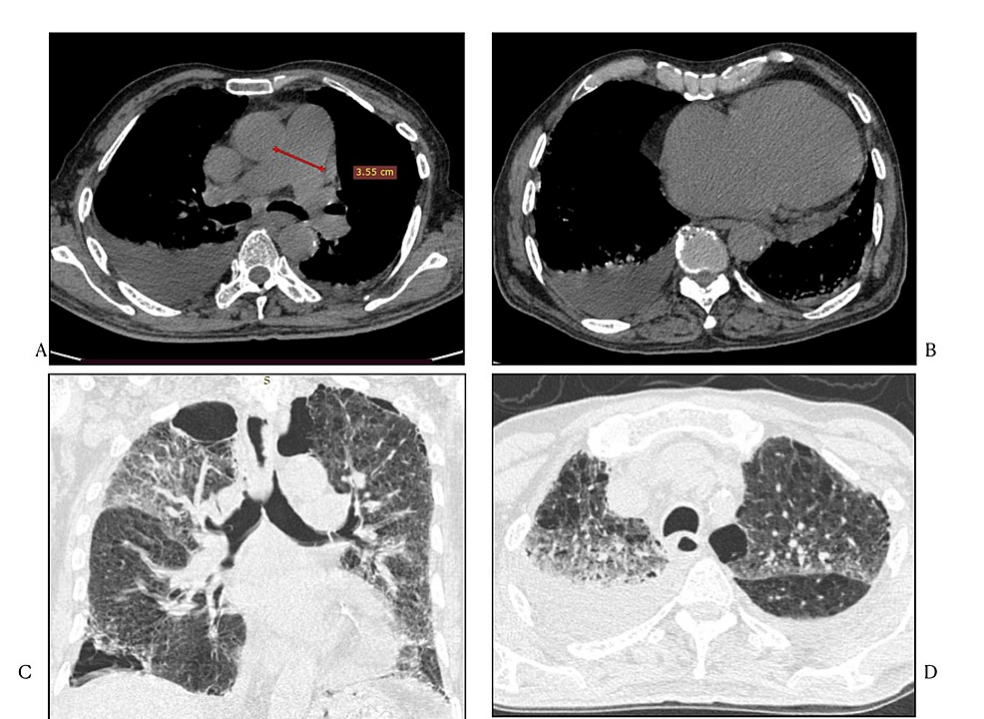
HRCT Chest (mediastinal and lung windows) in axial and coronal reformatted images A) Dilated main pulmonary artery (red line) and moderate right pleural effusion B) Cardiomegaly with minimal pericardial effusion C) & D) Peripheral ground glass opacities with background emphysematous changes

Another novel finding seen in our study was dilated main pulmonary artery seen in 12 patients, with at least 11 of them having imaging signs of pulmonary fibrosis (Figure 3).

**Figure 4:**
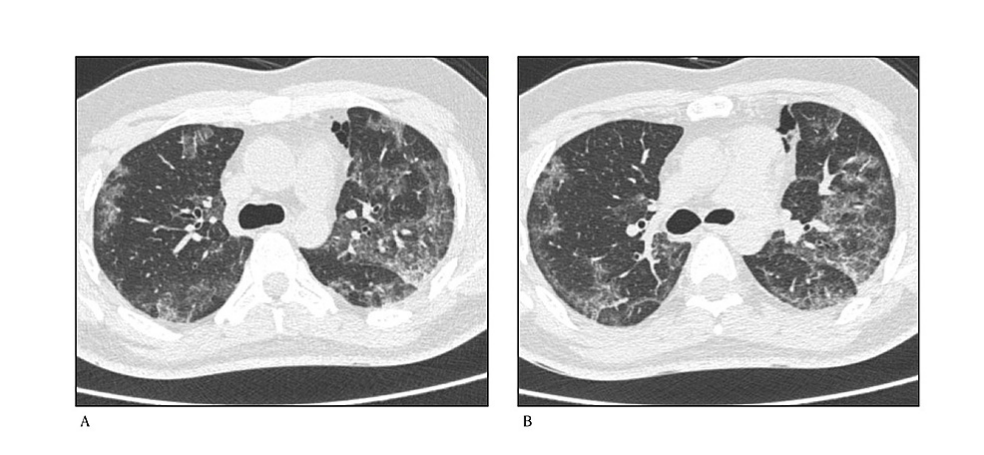
HRCT Chest lung window axial sections A) and B) Patchy multifocal (both peripheral and central distribution) ground glass opacities with areas of associated inter/intralobular septal thickening giving crazy paving pattern

The second most common finding in our study was ground glass opacities seen as patchy geographical areas of increased lung attenuation with total incidence of 82.85%, seen either in isolation or in association with interstitial thickening/consolidation. In studies by Salehi et al. [9] and Wang et al. [22], ground glass opacities were reported in 88% and 83 to 85%, respectively. GGO was described as the predominant pattern of abnormalities after symptom, while consolidation was the second most seen feature in the first 11 days. In our study the classical peripheral subpleural distribution was seen in 20 cases (28.57%). Additionally, pure ground glass opacities as the only imaging finding, which are considered hallmark of early disease process, was seen in 36 (51.42%) patients and total ground glass opacities (combination with other findings) was noted in total 58 (82.85%) cases. There was no lobar predominance with bilateral multifocal peripheral/sub-pleural distribution as the dominant pattern and crazy paving (Figure 4).

The other common findings described in previous literature are crazy paving pattern, organizing consolidation and reverse halo sign. In our study crazy paving was in 52.85%, organizing consolidation in 15.71% cases and reverse halo sign in 5.71% cases. 

Emerging recent data suggests that uncommon atypical pulmonary and extrapulmonary findings, like centrilobular nodules, cavitation, pleural/pericardial effusion, lymphadenopathy, pneumothorax/pneumomediastinum and pulmonary thromboembolism can be present and have been associated more in critically ill patients [9,16].

**Figure 5:**
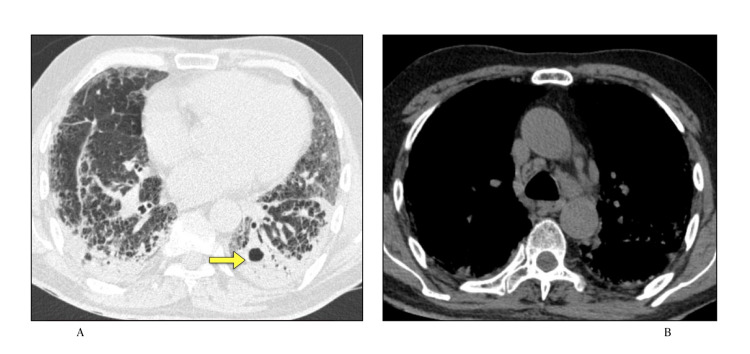
HRCT Chest (lung and mediastinal windows) A) Focal consolidation in left lower lobe with focal cystic changes (air bubble sign- shown by arrow) with background changes of fibrosis B) Enlarged mediastinal lymph nodes

In our study mediastinal lymph nodes (Figure 5) were seen in 24 (34.28%) cases and pleural effusion in nine (12.85%) and pericardial effusion in two (2.85%) cases. According to studies by Salehi et al. and Li et al. [9,16], these features are rarely present in the early stage of COVID-19 on chest CT, but it may be observed during disease progression. Further it was observed that severe and critical patients showed higher incidence of lymph nodes enlargement, pericardial effusion and pleural effusion than uncomplicated patients.

**Figure 6:**
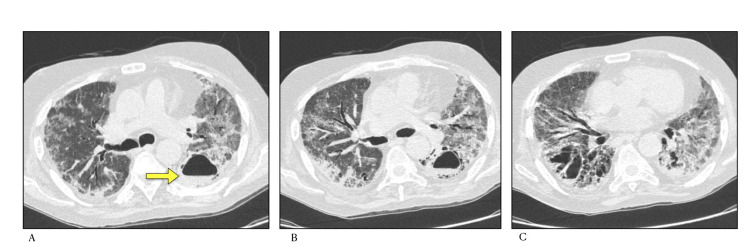
HRCT Chest (lung window) axial scans A), B) and C) demonstrate a cavity with an air fluid level in left lower lobe (arrow) with background changes of organizing consolidation and fibrosis

In our study, cavitation (Figure 6) was seen in four (5.71%) cases and all of them had CT severity score more than 20. As observed by Salehi et al., cavitation is the least common finding in COVID-19 and is visible especially in the later stages [9].

**Figure 7:**
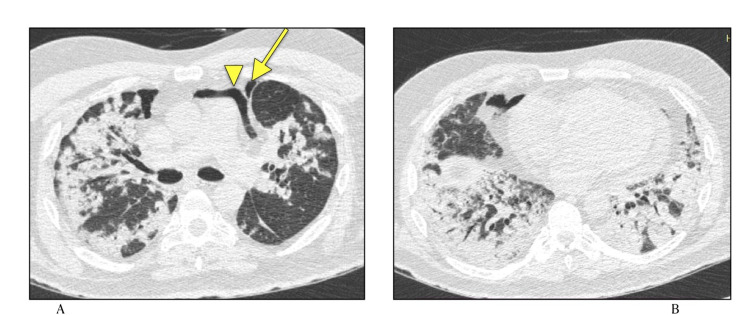
HRCT Chest (lung window) axial sections A) Pneumomediastinum (arrowhead) and pneumothorax (arrow) B) Areas of consolidation in bilateral upper lobes with few centrilobular nodules

Centrilobular nodules have also been infrequently seen [12]. In our study, they were observed in only one case, along with pneumomediastinum and pneumothorax (Figure 7).

**Figure 8:**
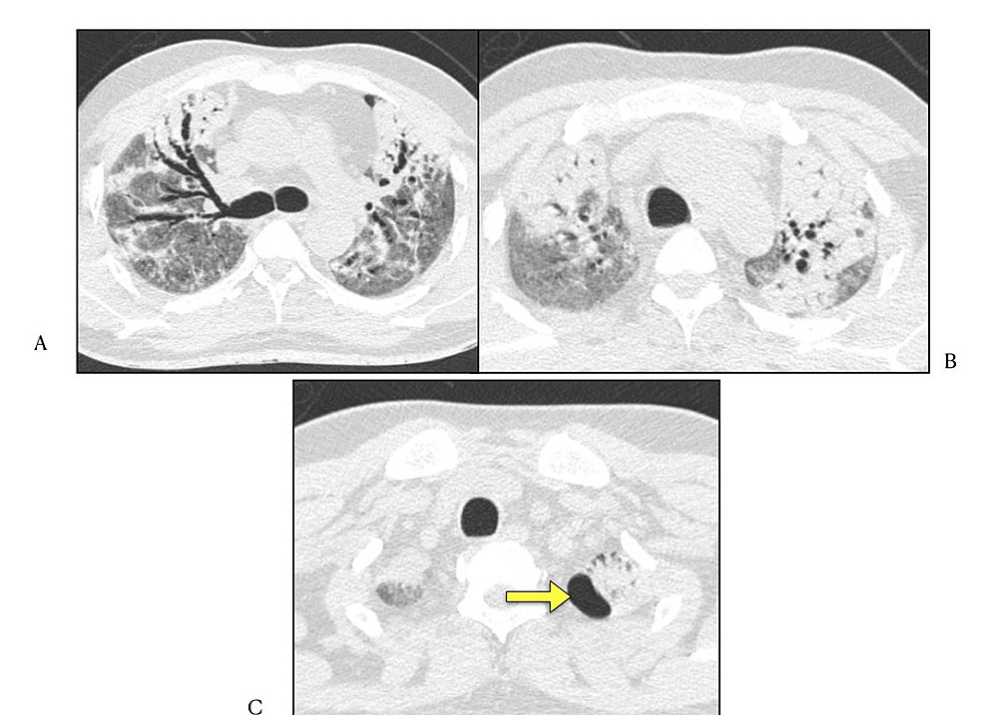
HRCT Chest (lung window) axial sections A) and B) Bilateral upper lobe consolidation with air bronchogram C) Cystic air space/pneumatocele/air bubble sign in left apico-posterior segment (arrow)

In our study cystic air spaces/air bubble sign/pneumatoceles (Figure 8) were reported in two (2.85%) cases. Pneumatocele is defined as spontaneously resolving newly formed cystic lesion in acute pneumonia [23]. In a study by Shi et al. lung cystic changes were seen in 10% of COVID-19 cases, although exact incidence and mechanism of pneumatocele formation is still unclear [24]. A Brazilian study [25] concluded incidence as high as 37% in their study.

Ruptured pneumatoceles can lead to pneumothorax [23]. Noteworthy risk factors for atraumatic pneumothorax mentioned in literature are prolonged pneumonitis, leucocytosis, severe clinical course and imaging features of fibrotic lung changes [26,27]. Another study concluded that pneumothorax is more common in mechanically ventilated patients, likely due to barotrauma. In our study one patient who had pneumothorax also had pneumomediastinum with high CT severity score and changes of fibrosis (Figure 7).

A case series by Reyes et al. [28] concluded that due to the extensive parenchymal damage caused by COVID-19, some patients may develop cystic changes, especially late into the course of the disease, which are at risk for rupture and subsequent pneumothorax even from the minimal trans pulmonary pressures generated from high flow nasal cannula.

Similarly spontaneous pneumomediastinum can also be seen in viral pneumonia. It has been hypothesized that damaged alveoli in severe COVID-19 pneumonia are prone to rupturing, especially in patients having pronounced cough. To date, there have been few reports on spontaneous pneumomediastinum from COVID-19 in the setting of non-mechanical ventilation [29]. In our study pneumomediastinum was seen in three (4.28%) cases.

**Figure 9:**
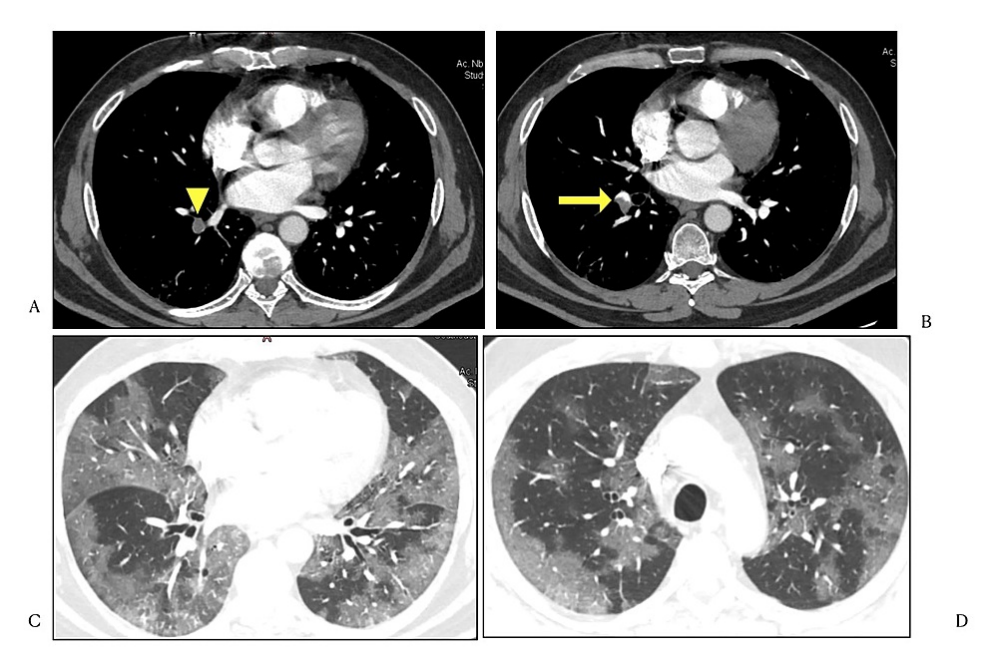
Contrast-enhanced CT Chest (mediastinal window and lung window) axial sections A) and B) show a hypodense filling defect in segmental pulmonary artery of right lower lobe (arrowhead and arrow), axial sections (lung window) show patchy multifocal ground glass opacities with both central and peripheral distribution

Pulmonary thromboembolism was previously considered as one of the rare complications of viral pneumonia. In our study, one patient had developed pulmonary thromboembolism (Figure 9). Bompard et al. [30] observed acute pulmonary thromboembolism in almost a quarter of the COVID-19 pneumonia patients evaluated after contrast administration on CT and concluded that contrast-enhanced CT should be more widely used, particularly in those with marked elevation of D-dimer.

## Conclusions

To conclude, in our study common and typical pulmonary findings like ground glass opacities, crazy paving pattern, organizing consolidation and reverse halo sign are in keeping with previous studies. However in contrast to previous many studies, the dominant finding in our study is early fibrosis which can be attributed to the fact that many patients underwent CT late in the course of illness; the average was 10-14 days of admission. Nonetheless, this supports the fact that disease progression in COVID-19 pneumonia is complicated by fibrosis. Emerging data in follow-up patients also corroborates our findings.

In our study, atypical pulmonary and extrapulmonary findings were seen in patients with high CT severity scores, which can be either due to heightened inflammatory response or secondary bacterial infection. But the fact is, less common but atypical findings are seen in complicated patients. So familiarity with both typical and atypical CT Chest Imaging findings is essential for a radiologist for better patient management.

However, the main limitation of our study was that true incidence rate could not be calculated as most of the patients in our cohort who underwent HRCT had moderate to severe disease. And secondly, many patients were lost to follow-up so the long-lasting effects of COVID-19 pneumonia couldn’t be studied in our research.
